# Tea consumption and the risk of five major cancers: a dose–response meta-analysis of prospective studies

**DOI:** 10.1186/1471-2407-14-197

**Published:** 2014-03-17

**Authors:** Feifei Yu, Zhichao Jin, Hong Jiang, Chun Xiang, Jianyuan Tang, Tuo Li, Jia He

**Affiliations:** 1 Department of Health Statistics, Second Military Medical University, 800 Xiangyin Road, Shanghai 200433, China; 2 Office of compliance and Development, Center for drug evaluation, China food and drug administration, Shanghai, China; 3 Department of Endocrinology, Changzheng Hospital, Second Military Medical University, Shanghai 200003, China

**Keywords:** Tea consumption, Dose–response, Meta-analysis, Cancer

## Abstract

**Background:**

We conducted a dose–response meta-analysis of prospective studies to summarize evidence of the association between tea consumption and the risk of breast, colorectal, liver, prostate, and stomach cancer.

**Methods:**

We searched PubMed and two other databases. Prospective studies that reported risk ratios (RRs) with 95% confidence intervals (CIs) of cancer risk for ≥3 categories of tea consumption were included. We estimated an overall RR with 95% CI for an increase of three cups/day of tea consumption, and, usingrestricted cubic splines, we examined a nonlinear association between tea consumption and cancer risk.

**Results:**

Forty-one prospective studies, with a total of 3,027,702 participants and 49,103 cancer cases, were included. From the pooled overall RRs, no inverse association between tea consumption and risk of five major cancers was observed. However, subgroup analysis showed that increase in consumption of three cups of black tea per day was a significant risk factor for breast cancer (RR, 1.18; 95% CI, 1.05-1.32).

**Conclusion:**

Ourresults did not show a protective role of tea in five major cancers. Additional large prospective cohort studies are needed to make a convincing case for associations.

## Background

Tea is a popular beverage consumed worldwide, generally in the forms of black and green tea. Tea is produced from the leaves of the *Camellia sinensis* plant through several processes. Black tea is the main tea beverage in the United States, Europe, and Western Asia, while green tea is more popular in China, Japan, and Korea [1]. Extensive laboratory studies using multiple animal models have suggested that tea and tea polyphenols might have an inverse association with cancer through its apoptosis-inducing, anti-mutagenic, and antioxidant properties [2,3].

In some recent reviews, researchers have suggested that green tea, which contains abundant polyphenols and catechins, specifically epigallocatechin-3-gallate (EGCG) 5, might have a protective effect against cancers. Using multiple approaches, studies have shown that polyphenols, theaflavins (TF) and thearubigins (TR) in black tea might possess chemopreventive properties. However, most of the evidence showing a protective effect of tea on cancer has been generated in animal experiments but has not been demonstrated in human trials [4,5].

The World Cancer Research Fund report of 2007 concluded that the evidence for associations between the consumption of tea and risk of some major cancers was still limited and inconsistent [6]. The results from a few clinical trials and epidemiological studies also indicated that the preventive effect of tea or its extract on cancer is controversial. In a recent clinical trial evaluating the efficacy of green tea extract (GTE) on prostate cancer, it was found that the GTE had minimal clinical activity [7,8]. However, another Phase II clinical trial suggested that higher doses of GTE might improve the short-term outcome in patients with a higher risk of oral premalignant lesions [9]. In a cohort study conducted in the USA, tea consumption was found to have no inverse association with colorectal cancer, and the hazard ratio (HR) changed only slightly as tea consumption increased [10]. However, in another cohort study conducted in China, results showed that regular green tea consumption was associated with a reduced risk of colorectal cancer in smokers [11]. Some recent systematic reviews have revealed conflicting results between meta-analyses with prospective studies and those with retrospective studies [12-16].

Previous meta-analyses mainly focused on the relationship between the highest tea consumption level and either the lowest tea consumption level or non-drinkers. However, the range of tea consumption and the cut-offs for the categories differed between studies. Another drawback of the previous meta-analyses is the inclusion of retrospective case–control studies, which were sensitive to confounding variables and bias, especially recall bias. To quantitatively assess the relationship between tea consumption and risk of five major cancers, we conducted a systematic review and dose–response meta-analysis with prospective studies. The five major cancers we studied were liver, stomach, breast, prostate, and colorectal cancer.

## Methods

### Literature search

We performed a systematic literature search in PubMed, Embase, and Cochrane Library with a combination of the following terms: tea AND (breast OR prostate OR stomach OR gastric OR colorectal OR colorectum OR rectal OR rectum OR colon OR large bowel OR liver OR hepatic OR hepatoma) AND (cancer OR cancers OR carcinoma OR carcinomas OR neoplasm OR neoplasms). No language restrictions were imposed. Reference lists of the identified publications were also reviewed for inclusion/exclusion. We also searched the conference abstract on the website of American Society of Clinical Oncology (ASCO) annual meeting from 2004 to 2013. Two reviewers (FY and CX) independently selected studies based on the titles and abstracts of the retrieved studies. Studies were included if they met the following criteria: (1) a prospective study assessing the association between tea consumption and at least one of the five selected cancers (breast, stomach, colorectal, liver and prostate cancer); (2) a study considering at least three levels of tea consumption and providing a sample size for cases and non-cases in each exposure category [17]; and (3) a study reporting the relative risks (RRs) of different dose categories with 95% confidence intervals (95% CIs) adjusted for sex, age, or other factors. We excluded retrospective case–control studies because of their inherent limitations, especially recall bias. However, nested case–control studies and case-cohort studies were included in our meta-analysis because the at-risk study populations in each of the exposure categories are derived from cohort studies and diet exposure would have been investigated years before the onset of cancer, which would technically eliminate recall bias. We also excluded studies that reported total tea consumption on monthly, weekly, or daily basis, but did not provide data on number of cups or times per month, week or day. One study assessing breast cancer risk was excluded because we could not extract data stratified by sex in both exposed and unexposed groups [18]. Another study was excluded because it reported stomach cancer risk based on iced tea and hot tea consumption, but no other study provided such data [19].

### Data extraction

Data extraction was performed according to the MOOSE (meta-analysis of observation studies in epidemiology) guidelines [20] (see the Additional file 1) by two reviewers (FY and CX), and verified independently for accuracy by a third reviewer (ZJ). Discrepancies were discussed with a fourth reviewer (JH) to reach a consensus. For each included study, the following data was extracted: the title and author of the study, publication year, study population, study location, sample size, proportion of males, median of follow-up time, the type of consumed tea, covariates controlled for by matching or multivariable analysis, the numbers of cases/non-cases, total person-years, relative risk (RR) of the different exposure categories and the corresponding 95% confidence intervals (95% CIs), response rate, and how exposure were assessed. For studies that reported several multivariable adjusted RRs, we selected the effect estimate that adjusted for the maximum potential confounders. The quality of the included studies was assessed according to the 9-star Newcastle-Ottawa Scale (NOS) [21] by two investigators (FY and HJ).

### Statistical analysis

We performed a two-stage dose–response meta-analysis to examine the relationship between tea consumption and five major cancer risks. First, we synthesized the RRs across categories of tea consumption in each study [22,23]. Because the absolute risk of cancer is low, the odds ratios (ORs) in nested case–control studies approximated the RRs [24]. Pooling of RRs from each study requires the exposure levels and distribution of cases and person-years or non-cases in each category of tea consumption. However, not all studies reported the distributions of cases and person-years or non-cases for exposure categories. Nine studies did not report individuals or person-years for each category and instead reported the total sample size [10,11,25-31]. We estimated the distribution of cases for each category in such studies by using the methods described by Aune [32]. To assess exposure levels, we converted all measures into cups per day and defined 125 mL of tea as one cup regardless of tea type unless it was well established in a specific study population or a geographical area. If the study reported tea consumption as number of times, we regarded one time as one cup. As some Chinese studies reported the amount of tea leaves consumed as the measure of tea consumption, we regarded consumption of 150 g of tea leaves per month as one cup per day; this allowed us to universalize all of the included studies in a single standard unit [33].

If a study did not report the median of the exposure category, we assigned the level of tea consumption to categories based on the calculated midpoint of tea consumption. When the highest category was open-ended, we assumed the dose as 1.2 times the lowest bound of this category [17]. In studies reporting tea consumption by cups per month or cups per week, we redefined these exposure categories as cups per day by multiplying with 1/30 or 1/7, respectively. Subsequently, we estimated the overall RR by combining the RRs derived from the first step. A fixed effect model was used if there was no evidence of heterogeneity; otherwise a random effect model was adopted [34,35]. Forest plots were used to visually assess the RR estimates and corresponding 95% CIs. We also tested the nonlinear relationship between tea consumption and cancer risk by modeling tea consumption levels by using restricted cubic splines with 3 knots at fixed percentiles (10%, 50%, and 90%) of the distribution as described by Larsson and Orsini [36,37]. A *P* value for nonlinearity was calculated by testing the null hypothesis that the coefficient of the second and third spline was equal to zero.

Eight studies on colorectal cancer [10,26,27,31,38-41] and three studies on stomach cancer [27,42,43] reported cancer risk by sex. Ten studies reported colorectal cancer risk stratified as colon or rectal cancer [10,18,26,27,38-41,44,45]. One study reported colon cancer risk as a distal or proximal cancer [38]. The study by Inoue *et al*. pooled data from six cohort Japanese studies that studied the relationship between tea consumption and stomach cancer [43]. Results from these studies were first pooled by using a fixed model and then included in the overall risk estimate. The χ^2^ test and I^2^ statistic were used to explore the heterogeneity among studies [46]. The Egger’s regression test, Begg’s rank correlation test, and visual inspection of a funnel plot were performed to assess publication bias [47,48]. As a rule of thumb, tests for asymmetry should be used only when there are at least 10 studies included in a meta-analysis [35]. We conducted subgroup analyses stratified by sex, tea types, and geographic regions. For breast cancer, we also performed a subgroup analysis stratified by menopausal status. We performed a sensitivity analysis in which one study at a time was removed and the rest analyzed to evaluate whether the results could have been affected markedly by a single study. To detect whether different assessment ways may bias the results, further subgroup analyses were performed by excluding studies that reported tea consumption by frequency (times/servings) [18,42,49-52], weight of tea leaves (grams) [11,27,33], or volume (mL) [53,54], rather than cups.

We used Stata (Version 12.0; Stata Corp, College Station, TX) for all analyses and all statistical tests were two-sided. P < 0.05 was considered statistically significant.

## Result

### Study characteristics

**Figure 1:**
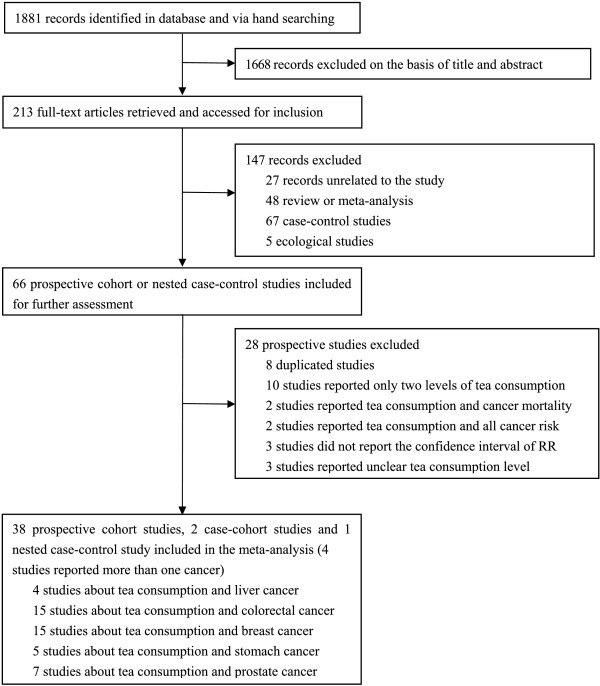
Summary of article selection process.

As of December 28, 2013, 1,881 records were retrieved by using our search strategy. After reviewing the titles and abstracts, we excluded 1,668 articles and 213 articles were further evaluated by reviewing the full texts. Finally, we identified 41 articles assessing tea consumption and cancer risk, which satisfied the inclusion criteria for our meta-analysis. A flow diagram of study selection is provided as Figure 1. Among the 41 articles, 15 assessed the relationship between tea drinking and the incidence for breast cancer [25,33,41,49,51,54-63], 15 for colorectal cancer [10,11,18,26-29,31,38-41,44,45],[59], 4 for liver cancer [18,27,64,65], 7 for prostate cancer [30,50,53,66-69], and 5 for stomach cancer [27,42,43,52,59]. The 41 articles included had 3,027,702 participants and 49,103 cancer cases. The cancer cases included 20,500 breast cancer patients; 16,202 colorectal cancer patients; 882 liver cancer patients; 4,698 prostate cancer patients; and 6,821 stomach cancer patients. Most of the included studies awarded more than 7 stars according to the Newcastle-Ottawa Scale and were identified as high quality. The general characteristics of the included studies are presented in Table 1.

**Table 1:** **Main characteristics of the studies on tea consumption and five selected cancer included in the meta**-**analysis**

**Reference**	**Country**	**Study design**	**Sampling**	**Tea type**	**Number of participants**	**Number of cases**	**Age**	**Follow-up(year)**	**Male (%)**
**Breast Cancer**									
Fagherazzi *et al*. 2011 [57]	France	Cohort	Population based (E3N study)	Tea (unclear)	67703	2868	40-65	11	0 (0)
Iwasaki *et al*. 2010 [60]	Japan	Cohort	Population based (JPHC study)	Green tea & Black tea	53793	845	40-69	13.6	0 (0)
Dai *et al*. 2010 [33]	China	Cohort	Population based (SWHS study)	Green tea	72861	614	40-70	7.3	0 (0)
Boggs *et al*. 2010 [56]	USA	Cohort	Population based (BWHS study)	Tea (unclear)	52062	1268	21-69	12	0 (0)
Pathy *et al*. 2010 [55]	Dutch	Cohort	Population based (EPIC-NL study)	Tea (unclear)	27323	681	20-70	9.6	0 (0)
Larsson *et al*. 2009 [61]	Sweden	Cohort	Population based (Swedish Mammography Cohort)	Black tea	66651	2952	40-76	17.4	0 (0)
Ishitani *et al*. 2008 [25]	USA	Cohort	Population based (Women’s Health Study)	Tea (unclear)	38432	1188	>45	10	0 (0)
Ganmaa *et al*. 2008 [58]	USA	Cohort	Registered nurses (Nurses’ Health Study)	Tea (unclear)	85987	5272	30-55	22	0 (0)
Hirvonen *et al*. 2006 [54]	France	Cohort	Double-blind placebo-controlled primary-prevention trial (SU.VI.MAX Study)	Tea (unclear)	4396	95	35-60	6.6	0 (0)
Adebamowo *et al*. 2005 [49]	USA	Cohort	Registered nurses (Nurses’ Health Study II)	Tea (unclear)	90638	710	25-46	4	0 (0)
Suzuki *et al*. 2004 [63]	Japan	Cohort	Population based	Green tea	35004	222	40-64	7-9	0 (0)
Michels *et al*. 2002 [62]	Sweden	Cohort	Population based (Swedish Mammography Screening Cohort)	Tea (unclear)	59036	1271	40-76	10.8	0 (0)
Key *et al*. 1999 [51]	Japan	Cohort	Hiroshima or Nagasaki bombings survivor (LSS study)	Green tea & Black tea	34759	405	<40 to >80	1969-1993	0 (0)
Zheng *et al*. 1996 [41]	USA	Cohort	Population based (Iowa Women’sHealth Study)	Non-herbal tea	35369	1602	55-69	8	0 (0)
Goldbohm *et al*. 1996 [59]	Netherlands	Case-cohort	Population based (Netherlands Cohort Study on Diet and Cancer)	Black tea	1376	507	55-69	4.3	0 (0)
**Colorectal Cancer**									
Dominianni *et al*. 2013 [29]	USA	Cohort	Population based (The PLCO Cancer Screening Trial)	Tea	57398	683	55-74	11.4	27596 (48.1)
Sinha *et al*. 2012 [10]	USA	Cohort	Population based (NIH-AARP Study)	Tea (unclear)	489706	6946	50-71	10.5	292211 (59.7)
Yang *et al*. 2011 [11]	China	Cohort	Population based (SMHS Study)	Green tea	60567	243	40-74	4.6	60567 (100)
Simons *et al*. 2010 [38]	Netherlands	Case-cohort	Population based (NLCS Study)	Tea (unclear)	3877	2199	55-69	13.3	58279 (48.2)
Lee *et al*. 2007 [26]	Japan	Cohort	Population based (JPHC study)	Green tea	96162	1163	52.1	10	46023(47.9)
Oba *et al*. 2006 [31]	Japan	Cohort	Population based (Cohort in Takayama)	Green tea	30221	213	>35	1992-2000	13894 (46.0)
Michels *et al*. 2005 [39]	USA	Cohort	Registered nurses and health professionals (NHS and HPFS)	Tea (unclear)	133893	1402	30-75	18 and 12	46099 (34.4)
Suzuki *et al*. 2005 [44]	Japan	Cohort	Population based	Green tea	26311	269	40-64	8-9	-
Su *et al*. 2002 [28]	USA	Cohort	Population based (NHEFS study)	Tea (unclear)	10011	219	25-74	20	-
Terry *et al*. 2001 [45]	Sweden	Cohort	Population based (The Swedish Mammography Screening Cohort)	Black tea	61463	460	40-76	9.6	0 (0)
Nagano *et al*. 2001 [18]	Japan	Cohort	Atomic bomb survivor (LSS study)	Green tea	38540	596	55.3	16	14873 (38.6)
Hartman *et al*. 1998 [40]	Finnish	RCT	Randomized, double-blind, placebo-controlled prevention trial (ATBC Study)	Tea (unclear)	27029	185	57.2	6.1	27111 (100)
Zheng *et al*. 1996 [41]	USA	Cohort	Population based (Iowa Women’s Health Study)	Non-herbal tea	35369	474	55-69	8	0 (0)
Goldbohm *et al*. 1996 [59]	Netherlands	Case-cohort	Population based (Netherlands Cohort Study on Diet and Cancer)	Black tea	2929	564	55-69	4.3	0 (0)
Nechuta *et al*. 2012 [27]	China	Cohort	Population based (Shanghai Women’s Health Study)	Tea (any)	69310	586	40-70	11	0 (0)
**Liver Cancer**									
Nechuta *et al*. 2012 [27]	China	Cohort	Population based (Shanghai Women’s Health Study)	Tea (any)	69310	134	40-70	11	0 (0)
Ui *et al*. 2009 [65]	Japan	Cohort	Population based (Ohsaki Cohort study)	Green tea	41761	247	40-79	9	19748 (47.3)
Inoue *et al*. 2009 [64]	Japan	Cohort	Population based (Japan Public Health Center-Based Prospective Study Cohort II)	Green tea	18815	110	40-69	12.7	6420 (34.1)
Nagano *et al*. 2001 [18]	Japan	Cohort	Atomic bomb survivor (LSS study)	Green tea	38540	391	55.3	16	14873 (38.6)
**Prostate Cancer**									
Geybels *et al*. 2013 [69]	Netherlands	Case-cohort	Population based (The Netherlands Cohort Study)	Black tea	5490	3362	55-69	17.3	5490 (100)
Montague *et al*. 2012 [30]	Singepore	Cohort	Population based (Singapore Chinese Health Study)	Green tea & Black tea	27293	298	45-74	11.2	27293 (100)
Shafique *et al*. 2012 [68]	Canada	Cohort	Employed men and women (Collaborative Cohort Study)	Tea (unclear)	6016	186	21-75	28	6016 (100)
Kurahashi *et al*. 2008 [67]	Japan	Cohort	Population based (Singapore Chinese Health Study)	Green tea	49920	404	40-69	15	49920 (100)
Kikuchi *et al*. 2006 [66]	Japan	Cohort	Population based (Ohsaki Cohort Study)	Green tea	18961	110	40-79	7	18961 (100)
Allen *et al*. 2004 [50]	Japan	Cohort	Atomic-Bomb Survivors (LSS Study)	Green tea & Black tea	18115	193	18-99	16.9	18115 (100)
Ellision *et al*. 2000 [53]	Canada	Cohort	Population based (NCS Study)	Tea (unclear)	3400	145	50-84	20	3400 (100)
**Stomach Cancer**									
Nechuta *et al*. 2012 [27]	China	Cohort	Population based (Shanghai Women’s Health Study)	Tea (any)	69310	293	40-70	11	0 (0)
Inoue *et al*. 2009 [43]	Japan	Cohort	Pooled Study (JPHC-I, JPHC-II, JACC, MIYAGI,3-pref MIYAGI,3-pref AICHI)	Green tea	219080	3577	40-103	8-15	100479 (45.9)
Sauvaget *et al*. 2005 [52]	Japan	Cohort	Atomic-Bomb Survivors (LSS Study)	Green tea	38576	1270	34-98	1980-1999	14885 (38.6)
Galanis *et al*. 1998 [42]	Japan	Cohort	Population based	Green tea	11907	108	46.4	14.8	5610 (47.1)
Goldbohm *et al*. 1996 [59]	Netherlands	Case-cohort	Population based (Netherlands Cohort Study on Diet and Cancer)	Black tea	2929	182	55-69	4.3	0 (0)
**Reference**	**Response rate**	**Assessment of exposure**	**Adjustments**						**Quality score (NOS stars)**
**Breast Cancer**									
Fagherazzi *et al*. 2011 [57]	UK	Self-administered FFQ	Total energy intake, ever use of oral contraceptives, age at menarche, age at menopause, number of children, age at first pregnancy, history of breast cancer in the family and years of schooling, current use of postmenopausal hormone therapy, personal history of benign breast disease, menopausal status and BMI						7
Iwasaki *et al*. 2010 [60]	>80%	Self-administered FFQ	Age, area, age at menarche, menopausal status at baseline, number of births, age at first birth, height, BMI, alcohol intake, smoking status, leisure time physical activity, daily physical activity, exogenous hormone use, family history of breast cancer, oolong tea intake, black tea intake, coffee intake, canned coffee intake and Sencha and Bancha/Genmaicha intake.						8
Dai *et al*. 2010 [33]	92%	In-person interview (frequency of tea consumption)	Age, educational achievement, income, family history of breast cancer, history of fibro adenoma, body mass index, waist-to-hip ratio, physically active, smoking status, alcohol consumption status, passive smoking status, ginseng intake, age at menarche, age at first live birth, menopausal status, age at menopause, use of hormone replacement therapy, and dietary intake of total energy, fruits, vegetables, red meat, fish, and isoflavones.						9
Boggs *et al*. 2010 [56]	>80%	Self-administered FFQ	Age, energy intake, age at menarche, BMI at age 18, family history of breast cancer, education, geographic region, parity, age at first birth, oral contraceptive use, menopausal status, age at menopause, female hormone use, vigorous activity, smoking status, alcohol intake, coffee and decaffeinated coffee						8
Pathy *et al*. 2010 [55]	UK	Self-administered FFQ	Propensity score (based on age, smoking status, educational status, BMI, alcohol intake, energy intake, energy adjusted saturated fat intake, energy adjusted fiber intake, coffee intake, physical activity level, ever use of oral contraceptives, presence of hypercholesterolemia, family history of breast cancer, age at menarche, parity, and cohort)						7
Larsson *et al*. 2009 [61]	74%	Self-administered FFQ	Age, education, body mass index, height, parity, age at first birth, age at menarche, age at menopause, use of oral contraceptives, use of postmenopausal hormones, family history of breast cancer, intakes of total energy, alcohol and coffee						7
Ishitani *et al*. 2008 [25]	100%	Self-administered FFQ	Age, randomized treatment assignment, body mass index, physical activity, total energy intake, alcohol intake, multivitamin use, age at menopause, age at menarche, age at first pregnancy lasting ≥6 months, number of pregnancies lasting ≥6 months, menopausal status, postmenopausal hormone use, prior hysterectomy, prior bilateral oophorectomy, smoking status, family history of breast cancer in mother or a sister, and history of benign breast disease						8
Ganmaa *et al*. 2008 [58]	90%	Self-administered FFQ	Age months, smoking status, body mass index, physical activity, height, alcohol intake, family history of breast cancer in mother or a sister, history of benign breast disease, menopausal status, age at menopause, use of hormone therapy, age at menarche, parity and age at first birth, weight change after18 and duration of postmenopausal hormone use and Coffee						7
Hirvonen *et al*. 2006 [54]	UK	Self-administered 24 h dietary record	Age, smoking, number of children, use of oral contraception, family history of breast cancer, and menopausal status						7
Adebamowo *et al*. 2005 [49]	>90%	Self-administered FFQ	Age at menarche, parity, age at first birth, family history of breast cancer in mother and/or sister, history of benign breast disease, oral contraceptive use, alcohol consumption, energy intake, current body mass index, height, smoking habit, physical activity and menopausal status						7
Suzuki *et al*. 2004 [63]	94%	Self-administered FFQ	Age, types of health insurance, age at menarche, menopausal status, age at first birth, parity, mother’s history of breast cancer, smoking, alcohol drinking, body mass index and consumption frequencies of black tea and coffee						8
Michels *et al*. 2002 [62]	76%	Self-administered FFQ	Age, family history of breast cancer, height, body mass index, education, parity, age at first birth, alcohol consumption, total caloric intake						7
Key *et al*. 1999 [51]	53.4%	Self-administered FFQ	Attained age, calendar period, city, age at time of bombing and radiation dose						6
Zheng *et al*. 1996 [41]	42.3%	Self-administered FFQ	Age, education, smoking status, pack-years of smoking, physical activity, all fruit and vegetable Intake, waist/hip ratio, and family history of cancer, age at menarche, age at menopause, age at first pregnancy						7
Goldbohm *et al*. 1996 [59]	UK	Self-administered FFQ	Benign breast disease, history of breast cancer in mother and sisters, age at menarche, age at menopause, use of oral contraceptives, age atfirst birth, parity, body mass index, smoking status, education, and intakes of energy, fat, and alcohol						7
**Colorectal Cancer**									
Dominianni *et al*. 2013 [29]	78%	Self-administered FFQ	Age, gender, race, family history of colorectal cancer, education, body mass index, physical activity, smoking status, NSAID intake, history of diabetes, number of colorectal examinations up to 3 years before the start of study, hormone use, fruit intake, vegetable intake, meat intake, alcohol intake and study centre.						7
Sinha *et al*. 2012 [10]	UK	Self-administered FFQ	Age, sex, race, education, smoking status, time since quitting for former smokers, smoking dose, ever smoke a pipe or cigar, diabetes, colorectal screening, family history of colorectal cancer, regular non-steroidal anti-inflammatory drug use, marital status, BMI, frequency of vigorous physical activity, calories, fruit and vegetables, red meat, dietary calcium intake, alcohol, and menopausal hormone therapy in women						7
Yang *et al*. 2011 [11]	74.1%	In-person interview (frequency of tea consumption)	Age, education, cigarette smoking, pack-years of cigarette smoking, alcohol consumption, regular exercise, body mass index, history ofdiabetes, family history of colorectal cancer and intakes of vegetables, fruits and red meat						8
Simons *et al*. 2010 [38]	UK	Self-administered FFQ	Age, family history of CRC, non-occupational physical activity, smoking status, educational level, body mass index, ethanol intake, meat intake, processed meat intake, foliate intake, vitamin B6 intake, fiber intake, and fluid intake from other fluids						7
Lee *et al*. 2007 [26]	79%	Self-administered FFQ	BMI, smoking status, alcohol drinking, family history of colorectal cancer, physical activity, and intake of green vegetables, beef, pork, green tea, Chinese tea and black tea						7
Oba *et al*. 2006 [31]	92%	Self-administered FFQ	Age, height, BMI, total pack-years of cigarette smoking, alcohol intake, physical activity, black tea intake and green tea/coffee intake.						8
Suzuki *et al*. 2005 [44]	91.7%	Self-administered FFQ	Sex, age, family history of colorectal cancer, cigarette smoking, alcohol consumption, body mass index, consumption of black tea, and coffee. Cohort1 adjusted for consumption of meat, green-yellow vegetables, other vegetables, and fruits. Cohort2 adjusted for consumption of beef, pork, ham, chicken, liver, spinach, carrot or pumpkin, tomato, orange, other fruits, and juice						8
Michels *et al*. 2005 [39]	100% and 96%	Self-administered FFQ	Age, family history of colorectal cancer, history of sigmoidoscopy, height, body mass index, pack-years of smoking, physical activity, aspirin use, vitamin supplement intake, alcohol consumption, red meat consumption, total caloric intake, and, among women in addition for menopausal status, postmenopausal hormone use.						7
Su *et al*. 2002 [28]	92.2%	In-person interviews (24 h food recall)	Baseline age, race, education level, BMI, aspirin use, dietary intakes of calories, fat, fiber and calcium, and alcohol use at baseline.						9
Terry *et al*. 2001 [45]	98%	Self-administered FFQ	Age in 5-yr age groups, body mass index (quartiles), education level (3 categories), quartiles of total calories, red meat, coffee, alcohol, energy-adjusted total fat, fruit fiber, vegetable fiber, cereal fiber, calcium, vitamin C, folic acid, and vitamin D.						8
Nagano *et al*. 2001 [18]	72%	Self-administered FFQ	City, age, gender, radiation exposure, smoking status, alcohol drinking, body mass index, education level, calendar time						6
Hartman *et al*. 1998 [40]	_	Self-administered FFQ	Age, intervention group, calcium, occupational physical activity, and BMI.						7
Zheng *et al*. 1996 [41]	42.3%	Self-administered FFQ	Age, education, smoking status, pack-years of smoking, physical activity, all fruit and vegetable Intake, waist/hip ratio, and family history of cancer						7
Goldbohm *et al*. 1996 [59]	96%	Self-administered FFQ	Benign breast disease, history of breast cancer in mother and sisters, age at menarche, age at menopause, use of oral contraceptives, age at first birth, parity, body mass index, smoking status, education, and intakes of energy, fat, and alcohol						8
Nechuta *et al*. 2012 [27]	99.8%	In-person interview, self-administered FFQ	age, marital status, education, occupation, BMI, exercise, fruit and vegetable intake, meat intake, diabetes, and family history of digestive system cancer						9
**Liver Cancer**									
Nechuta *et al*. 2012 [27]	99.8%	In-person interview, self-administered FFQ	age, marital status, education, occupation, BMI, exercise, fruit and vegetable intake, meat intake, diabetes, and family history of digestive system cancer						9
Ui *et al*. 2009 [65]	94.6%	Self-administered FFQ	Age, sex, alcohol consumption, smoking status, coffee consumption, vegetable consumption, dairy products consumption, fruit consumption, fish consumption, soybean consumption						8
Inoue *et al*. 2009 [64]	82%	Self-administered FFQ	Sex, age, area, smoking status, weekly ethanol intake, body mass index, history of diabetes mellitus, coffee consumption, green tea consumption, serum ALT level, HCV infection status, and HBV infection status						8
Nagano *et al*. 2001 [18]	72%	Self-administered FFQ	City, age, gender, radiation exposure, smoking status, alcohol drinking, body mass index, education level, calendar time						7
**Prostate Cancer**									
Geybels *et al*. 2013 [69]	96%	Self-administered FFQ	Age						8
Montague *et al*. 2012 [30]	UK	In-person Interview	Age, dialect group, interview year, education, body mass index and smoking history, green/black tea intake						8
Shafique *et al*. 2012 [68]	70%	Self-administered FFQ	Age, body mass index, smoking status, coffee consumption, alcohol intake, cholesterol level, systolic blood pressure, social class, and years of full-time education						7
Kurahashi *et al*. 2008 [67]	77%	Self-administered FFQ	Age, area, smoking status, alcohol consumption, body mass index, marital status, and coffee, black tea, and miso soup consumption, fruits, green or yellow vegetables, dairy food, soy food, and genistein consumption						7
Kikuchi *et al*. 2006 [66]	95%	Self-administered FFQ	Age, body mass index, alcohol consumption, smoking status, marital status, daily calorie intake, daily calcium intake, walking duration, consumption frequencies of black tea and coffee and consumption frequencies of fish						8
Allen *et al*. 2004 [50]	UK	Interview-based FFQ	Age, calendar period, city of residence, radiation dose and education level						7
Ellision *et al*. 2000 [53]	47%	In-person interviews (24 h food recall and one month food frequency)	Age, coffee, cola, total alcohol, beer, wine, spirits, smoking status, pack-years smoking, body mass index, highest education level attained, respondent status, intake of fiber, fat, calories.						8
**Stomach Cancer**									
Nechuta *et al*. 2012 [27]	99.8%	In-person interview, self-administered FFQ	Age, marital status, education, occupation, BMI, exercise, fruit and vegetable intake, meat intake, diabetes, and family history of digestive system cancer						9
Inoue *et al*. 2009 [43]	82%, 80%, 83%, 92%, 94%, 90%	Self-administered FFQ	Age, area, smoking, ethanol intake, rice intake, soy bean paste soup, and coffee intake, pickled vegetable intake and green–yellow vegetable intake						8
Sauvaget *et al*. 2005 [52]	72.5%	Self-administered FFQ	Sex, sex-specific age, city, radiation dose, sex-specific smoking habits, and education level.						6
Galanis *et al*. 1998 [42]	95%	Self-administered FFQ	Age, years of education, Japanese place of birth, and gender. Analyses among men were also adjusted for cigarette smoking and alcohol intake status						8
Goldbohm *et al*. 1996 [59]	72%	Self-administered FFQ	Benign breast disease, history of breast cancer in mother and sisters, age at menarche, age at menopause, use of oral contraceptives, age at first birth, parity, body mass index, smoking status, education, and intakes of energy, fat, and alcohol						7

*UK*: unknown; *FFQ*: food frequency questionnaire.

### Tea consumption and cancers

**Figure 2:**
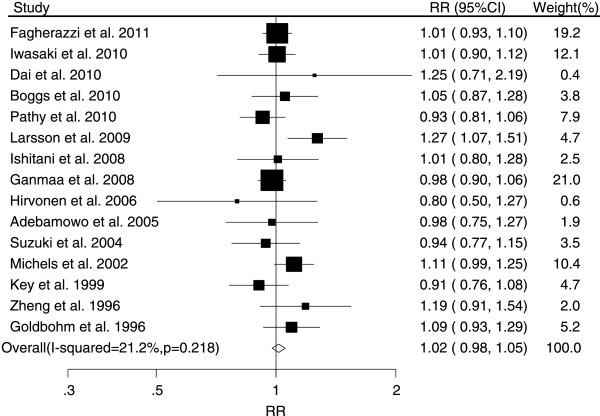
Relative risk estimates of breast cancer per 3 cups increase in tea consumption.

**Figure 3:**
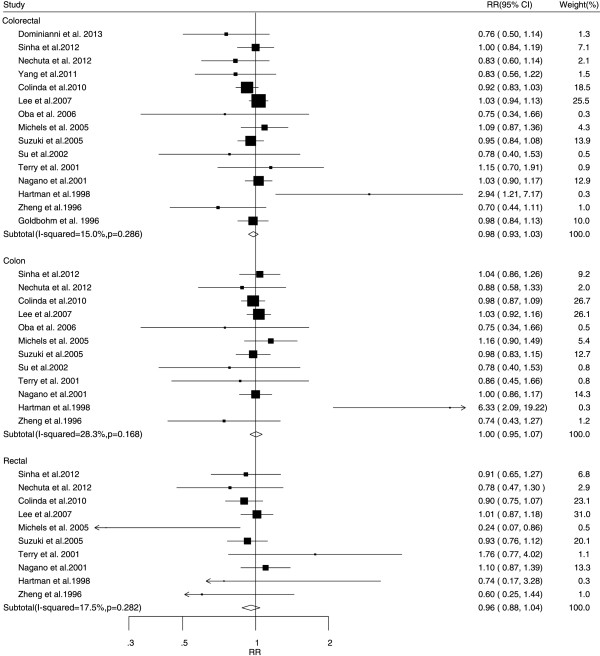
Relative risk estimates of colorectal cancer per 3 cups increase in tea consumption.

**Figure 4:**
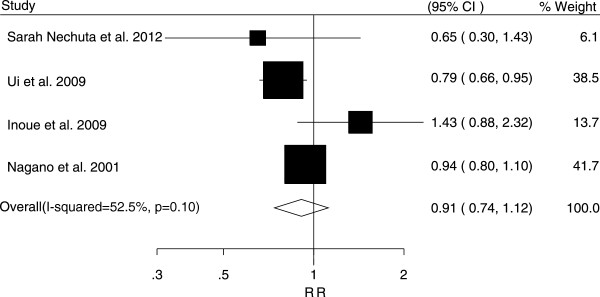
Relative risk estimates of liver cancer per 3 cups increase in tea consumption.

**Figure 5:**
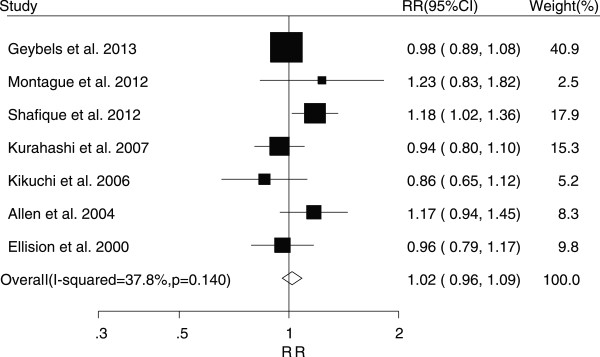
Relative risk estimates of prostate cancer per 3 cups increase in tea consumption.

**Figure 6:**
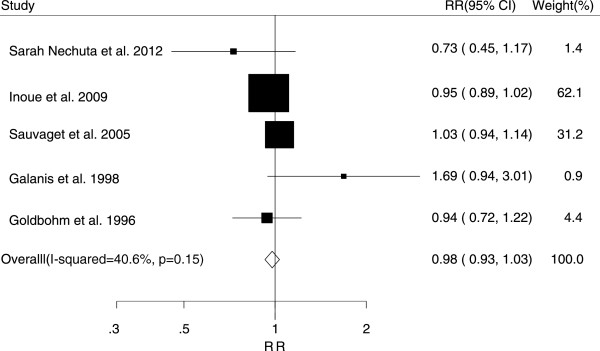
Relative risk estimates of stomach cancer per 3 cups increase in tea consumption.

The associations between tea consumption and the risk of major cancers are shown in Figures 2, 3, 4, 5 and 6. For breast cancer, the overall RR for three cups increment per day of tea consumption was 1.02 (95% CI, 0.98 to 1.05) with mild heterogeneity among studies (*P* = 0.22, *I*^2^ = 21.2%). For colorectal cancer, the pooled RR for three cups increment per day was 0.98 (95% CI, 0.93 to 1.03) with mild heterogeneity (*P* = 0.29, *I*^2^ = 15.0%). For liver cancer, the overall RR for three cups increment per day was 0.91 (95% CI, 0.74-1.12) with moderate heterogeneity (*P* = 0.10, *I*^2^ = 52.5%). For prostate cancer, the overall RR for three cups increment per day was 1.02 (95% CI, 0.96 to 1.09) with moderate heterogeneity (*P* = 0.14, *I*^2^ = 37.8%). For stomach cancer, the overall RR for three cups increment per day was 0.98 (95% CI, 0.93-1.03) with moderate heterogeneity (*P* = 0.15, *I*^2^ = 40.6%).

**Figure 7:**
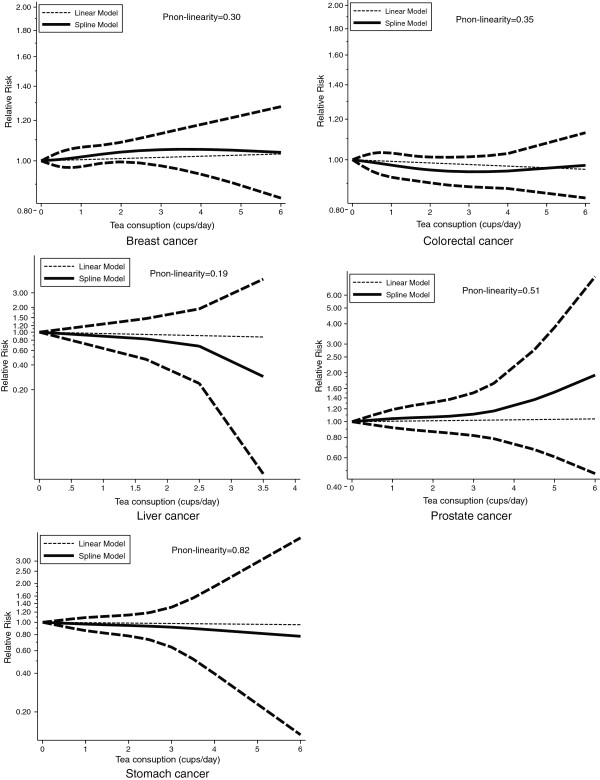
Dose–response relations between tea consumption and relative risks of breast, colorectal, stomach, prostate cancer.

As shown by Figure 7 and the P-value for nonlinearity, we found no evidence of nonlinear relationships between tea consumption and risk of cancers.

In the subgroup analysis, we pooled the studies into groups by sex, tea type, geographic region, and menopausal status for breast cancer. The results are shown in Table 2. We found that three cups of black tea consumption increment per day may be a risk factor for breast cancer (RR, 1.18; 95% CI, 1.05-1.32). The result of the subgroup analysis of stomach cancer indicated that tea consumption was a preventive factor (RR, 0.88; 95% CI, 0.80-0.98) in women. However, only three studies were included in this subgroup.

**Table 2:** **Subgroup analysis of cancer risk for an increment of three cup tea consumption by gender**, **tea type and geographic region**

**Subgroup**	**Breast**		**Stomach**		**Liver**		**Prostate**	
	***N***	**Pooled RR (95%CI)**	***N***	**Pooled RR (95%CI)**	***N***	**Pooled RR (95%CI)**	***N***	**Pooled RR (95%CI)**
By gender								
Male	-	-	2	1.20 (0.72-2.01)	-	-	6	1.02 (0.96-1.09)
Female	15	1.02 (0.98-1.05)	3	0.88 (0.80-0.98)	1	0.65 (1.30-1.43)	-	-
By menopausal status								
Pre-menopausal	2	0.96 (0.79-1.18)	-		-		-	
Post-menopausal	3	1.12 (0.96-1.30)	-		-		-	
By tea type								
Green tea	4	0.97 (0.90-1.06)	4	0.99 (0.94-1.05)	3	0.93 (0.75-1.17)	4	0.99 (0.88-1.11)
Black tea	4	1.18 (1.05-1.32)	1	0.94 (0.72-1.22)	-	-	3	0.99 (0.90-1.09)
By geographic region								
Europe	6	1.05 (0.96-1.15)	1	0.94 (0.72-1.22)	-	-	2	1.07 (0.90-1.27)
Asian	4	0.98 (0.90-1.06)	4	0.99 (0.87-1.12)	4	0.91 (0.74-1.12)	4	1.00 (0.90-1.12)
China	1	1.25 (0.71-2.19)	1	0.73 (0.45-1.17)	1	0.65 (0.30-1.43)	-	-
Japan	3	0.97 (0.89-1.05)	3	1.01 (0.86-1.14)	3	0.90 (0.80-1.01)	3	0.99 (0.88-1.11)
North America	5	1.00 (0.94-1.07)	-	-	-	-	1	0.96 (0.79-1.17)
**Subgroup**			**Colon**		**Rectal**			**Colorectal**
			***N***	**Pooled RR (95% CI)**	***N***	**Pooled RR (95% CI)**	***N***	**Pooled RR (95% CI)**
By gender								
Male			6	1.00 (0.90-1.11)	5	0.96 (0.83-1.11)	6	0.98 (0.90-1.07)
Female			6	1.02 (0.92-1.14)	2	0.93 (0.78-1.10)	6	0.99 (0.90-1.09)
By tea type								
Green tea			4	1.01 (0.93-1.09)	3	1.00 (0.90-1.12)	5	1.00 (0.94-1.07)
Black tea			1	0.86 (0.45-1.66)	1	1.76 (0.77-4.02)	2	0.99 (0.86-1.14)
By geographic region								
Europe			3	1.02 (0.84-1.24)	3	0.92 (0.77-1.10)	4	0.96 (0.88-1.04)
Asian			5	1.00 (0.93-1.08)	4	0.99 (0.89-1.10)	6	0.99 (0.93-1.06)
China			1	0.88 (0.58-1.33)	1	0.78 (0.47-1.30)	2	0.83 (0.64-1.06)
Japan			4	1.01 (0.93-1.09)	3	1.00 (0.90-1.12)	4	1.01 (0.94-1.07)
North America			4	1.04 (0.90-1.20)	3	0.81 (0.60-1.09)	5	0.97 (0.85-1.09)

A sensitivity analysis omitting one study at a time and calculating the pooled RRs for the remainder of the studies suggested that no single study dramatically influenced the pooled RRs (results are not shown). After removing the studies that did not report tea consumption as cups per day, the results did not change significantly. On excluding a study by Inoue *et al*. [43], which had a significantly larger sample size in comparison with other included studies, no significant differences were observed.

**Figure 8:**
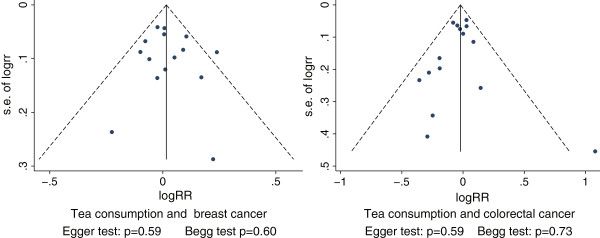
Funnel plot of log relative risk vs standard error of log relative risks.

Egger’s regression test and Begg’s rank correlation test showed no significant asymmetry of the funnel plot for breast (*P* = 0.59 and *P* = 0.60, respectively) and colorectal cancer (*P* = 0.59 and *P* = 0.73, respectively), indicating no evidence of substantial publication bias (Figure 8). For the other three types of cancer, we did not perform an analysis for publication bias because of limited numbers of included studies (no more than ten).

## Discussion

The findings from our meta-analysis reveal no appreciable association between tea consumption and the relative risk of liver, stomach, breast, prostate, or colorectal cancers. The risk differences were all near zero for the five major cancers with an increase in tea consumption of three cups per day (approximately 375 mL per day). Subgroup analyses, stratified by sex, geographic regions, and type of tea, showed no significant associations with risk of cancer.

The results of heterogeneity test suggested that most pooled effects had mild heterogeneity. However, moderate heterogeneity was seen in studies on liver cancer. Sensitivity analysis performed by omitting one study at a time, identified the main source of heterogeneity as the study by Inoue *et al*. [64], which included hepatitis C and B virus positive patients while the other three studies did not. After excluding this study, the overall RR was 0.86 (95% CI, 0.75-0.99) with a mild heterogeneity (*P* = 0.30, *I*^2^ = 17.1%), which suggests a preventive effect of tea drinking for liver cancer. However, large prospective studies are still needed to verify this preventive effect.

Eleven previous meta-analyses have evaluated the association between green tea or black tea intake and the risk of the five selected cancers [12-16,70-75]. Of these earlier meta-analyses, three focused on breast cancer, three on colorectal cancer, three on stomach cancer, one on primary liver cancer, and one on prostate cancer. All of the previous eleven meta-analyses reported the summarized RR/OR for the highest tea consumption level relative to the no tea/lowest level. In nine of the meta-analyses that examine the association between green tea consumption and cancer risk, three studies presented an overall statistically significant inverse association between green tea consumption and cancer risk when they included both cohort and case–control studies in the meta-analysis. The summarized ORs of these three studies were 0.82 (95% CI, 0.70-0.96) and 0.86 (95% CI, 0.74-1.00) for stomach cancer and 0.82 (95% CI, 0.69 -0.98) for colorectal cancer [71,72,75]. Among the eleven meta-analyses, seven studies reported an inverse association between green tea consumption and cancer risk from the summarized results of case–control studies. However, none of the studies indicated a statistically significant green tea-cancer risk association from a meta-analysis of prospective cohort studies only. These earlier results from prospective studies were consistent with our present meta-analysis. For a concise comparison, we have presented the pooled RRs of previous studies and our study in Table 3.

**Table 3:** **omparison of findings of the present dose**–**response meta**-**analysis with those reported in previous meta**-**analysis**

**Reference**	**Tea type**	Previous review*						**Present review**^**#**^		
		**Cohort study**		**Case–control study**		**Over all**				
		***N***	**Pooled RR (95%CI)**	***N***	**Pooled OR (95%CI)**	***N***	**Pooled RR (95%CI)**	**Tea type**	***N***	**Pooled RR (95%CI)**
**Breast cancer**										
Ogunleye *et al*. 2010	Green tea	2	0.85 (0.65-1.12)	5	0.81 (0.75-0.88)	7	0.81 (0.75-0.88)	Tea	15	1.02 (0.98-1.06)
Sun *et al*. 2006	Green tea	3	0.85 (0.66-1.09)	1	0.47 (0.26-0.85)	4	0.78 (0.61-0.98)	Green tea	4	0.97 (0.90-1.06)
Sun *et al*. 2006	Black tea	5	1.15 (1.02-1.31)	8	0.91 (0.84-0.98)	13	0.98 (0.88-1.09)	Black tea	4	1.18 (1.05-1.32)
Seely *et al*. 2005	Green tea	3	0.89 (0.71-1.10)	2	0.44 (0.14-1.31)	-	-			
**Colorectal cancer**										
Wang *et al*. 2012	Green tea	-	-	13	0.95 (0.81-1.11)	-	-	Tea	15	0.98 (0.93-1.03)
Sun *et al*. 2006	Green tea	4	0.97 (0.82-1.16)	4	0.74 (0.63-0.86)	8	0.82 (0.69-0.98)	Green tea	5	1.00 (0.94-1.07)
Sun *et al*. 2006	Black tea	7	1.02 (0.78-1.34)	13	0.98 (0.84-1.15)	20	0.99 (0.87-1.13)	Black tea	2	0.99 (0.86-1.14)
Zhang *et al*. 2010	Tea	13	1.28 (1.02-1.61)	-	-	13	1.28 (1.02-1.61)			
**Liver cancer**										
Sing *et al*. 2011	Tea	7	0.84 (0.69-1.02)	6	0.86 (0.44-1.14)	11	0.77 (0.57-1.03)	Tea Green tea	4	0.93 (0.75-1.17)
									3	0.93 (0.75-1.17)
**Prostate cancer**										
Zheng *et al*. 2011	Green tea	4	1.00 (0.66-1.53)	3	0.43 (0.25-0.73)	7	0.72 (0.45-1.15)	Tea	7	1.00 (0.87-1.15)
Zheng *et al*. 2011	Black tea	Prospective: 3	0.83 (0.63-1.08)	6	1.07 (0.78-1.48)	11	0.99 (0.82-1.20)	Green tea	4	0.99 (0.88-1.11)
		Retrospective: 2	1.04 (0.73-1.50)					Black tea	3	0.99 (0.90-1.09)
**Stomach cancer**										
Kang *et al*. 2010	Green tea	7	1.03 (0.92-1.16)	11	0.74 (0.63-0.86)	18	0.86 (0.74-1.00)	Tea	5	0.97 (0.92-1.02)
Myung *et al*. 2009	Green tea	7	1.04 (0.93-1.17)	8	0.73 (0.64-0.83)	15	0.82 (0.70-0.96)	Green tea	4	0.99 (0.94-1.05)
Zhou *et al*. 2008	Green tea	4	1.56 (0.93-2.60)	HCC:4	1.12 (0.70-1.77)	14	0.98 (0.77-1.24)	Black tea	1	0.94 (0.72-1.22)
				PCC:6	0.67 (0.49-0.92)					

*HCC*: Hospital based case–control *PCC*: population based case–control *N*: number of studies included.

*Pooled results between highest consumption level and non/lowest drinker.

^#^Pooled results with an increase in tea consumption.

Among the three meta-analyses examing the relationship between black tea consumption and cancer risk, Sun *et al*. presented conflicting results across cohort studies and case–control studies for breast cancer risk [73]. The pooled RR for prospective cohort studies was 1.15 (95% CI, 1.02 to 1.31), while the pooled RR for case–control studies was 0.91 (95% CI, 0.84 to 0.98). Our meta-analysis also found a positive association between black tea consumption and the risk of breast cancer in a subgroup analysis. The summarized RR was 1.18 (95% CI, 1.05-1.32) with an increment of three cups black tea intake per day. Association of black tea consumption with breast cancer risk is biologically plausible. Black tea intake has been positively associated the estrogen levels, and experimental studies have established that estrogen is a strong promoter of mammary carcinogenesis [76]. Wu *et al*. reported that the levels of circulating estrogens were higher in black tea drinkers than in non-tea drinkers [77], and Larsson *et al*. reported that black tea consumption was positively associated with the risk of ER+/PR + breast cancer, suggesting a possible carcinogenic role involving sex hormones [61].

Of the three meta-analyses exploring the association between tea consumption and colorectal cancer risk, Zhang *et al*. [74] found a positive association between tea and colorectal cancer (RR 1.28; 95% CI, 1.02-1.61) for tea consumption greater than four cups. Our pooled RR was 0.98 (95% CI, 0.93-1.03) with an increment of three cups black tea intake per day. These differing results could be attributed to the calculation using different increment cups per day. We concluded that drinking three cups more per day does not increase the risk of colorectal cancer while Zhang *et al*. concluded that drinking four cups increment per day might be a risk factor for colon cancer. In addition, Zhang *et al*. also reported that the risk of colon cancer did not increase with a 250 g/day increment of tea consumption either in men (RR, 1.03; 95% CI, 0.96-1.09) or women (RR, 1.03; 95% CI, 0.99-1.08); this is consistent with our current meta-analysis. Zhang *et al*. attributed the observed positive association to chance.

It is notable that most epidemiological evidence for the inverse association between tea intake and cancer risk has come from retrospective case–control studies. Because information in case–control studies is collected after cancer is diagnosed, it reflects the past exposure history based on recall; therefore, recall bias is inevitable and cannot be ignored. This bias may partly explain the difference in the findings between prospective studies and retrospective case–control studies. Given the limitations of case–control studies, the conclusion that tea consumption has an inverse association with cancer risk is not convincing.

When compared with previous meta-analyses, our meta-analysis has several strengths. First, we assessed the association of tea consumption with five major cancers. Second, only prospective studies were included in the meta-analysis, which greatly reduces the likelihood of selection and recall biases. Third, the dose–response analysis included a wide range of tea consumption, which allows a concrete and quantitative assessment of the dose–response relationship between tea intake and cancer risk.

However, several potential limitations of our meta-analysis must be considered when interpreting the results. First, the temperature of tea is an important confounding factor when assessing the association between tea drinking and cancer risk. However, we could not analyze the effect of this factor because only one study reported the relationship between iced or hot tea and cancer; this study reported no significant association were observed between iced or hot tea and gastric cancers. Second, the methods of data collection differed across the included studies. Most of the studies assessed the exposure of the tea consumption via food frequency questionnaire, and the response rate varied among these studies. As is known, people in different countries have different traditions of tea consumption or drinking style, which presents difficulties in assessing tea consumption amounts accurately. In addition, people from the same country may also have different habits of drinking tea, such as strong or weak tea. In addition, tea consumption level is mostly assessed as the number of cups of tea consumed daily or weekly. However, cup size may vary considerably for different countries or areas and the dry tea leaves brewed in each cup may also be different. The cut-offs for the highest consumption level varied across different studies. Therefore, there might be some inevitable measurement errors and possible uncontrolled confounding factors when assessing tea consumption; this could prevent the detection of a modest association between tea consumption and cancer risk. However, we endeavoured to decrease these errors by unifying the unit of measurement and using a similar standard to calculate daily tea consumption. We also performed sensitivity analyses by removing the studies that did not report tea consumption as cups and performed subgroup analyses by geographic region, especially China and Japan (see Table 2). Third, we had no information about the family history of certain cancers in the primary aggregate results. Genetic factors play an important role in the development of cancers. Some genetic factors may increase the susceptibility to cancer, and certain polymorphisms in genes that are responsible for metabolising tea may have a role in the tea-cancer association. Fourth, a meta-analysis is unable to account for confounding factors inherent in the original studies. Although major potential confounders, including age, sex, alcohol, and smoking had been adjusted in most included studies, residual or unknown confounding cannot be excluded as a potential explanation for the observed findings. It is known that alcohol and smoking are important potential confounding factors. For example, people in China who drink tea frequently are more likely to drink more alcohol or smoke. The interplay of these factors, tea, alcohol, and smoking, could not be detected because of limited data. Most of the included studies did not provide the numbers for case and non-case population at each level of tea consumption; therefore, based on the available data, analyses among non-smokers and non-alcohol drinkers could not be conducted. Additionally, not all studies adjusted for these confounding factors. For breast cancer, the menopausal status of women was an important confounding factor. However, according to our subgroup analysis on pre-menopausal (RR, 0.96; 95% CI, 0.79-1.18) and post-menopausal (RR, 1.12; 95% CI, 0.96-1.30) women, there were no significant risk associations between tea consumption and pre-menopausal or post-menopausal breast cancer. Considering only three studies were available for this subgroup analysis, the results may be because of limited sample size. Thus, menopausal status could be another effect modifier for breast cancer, and further studies are needed to discuss the confounding effect of this factor for breast cancer [33]. Finally, publication bias is an inevitable problem in systematic reviews and meta-analyses. However, the results of publication bias analysis showed that there was no significant evidence of publication bias in studies on breast and colorectal cancer. The limited number of included studies precludes us from conducting publication bias analyses for liver, prostate, or stomach cancer.

## Conclusion

There is insufficient information from epidemiologic studies to support the suggestion that tea intake plays a role in the prevention of cancer. Randomized controlled trials and large prospective cohort studies are needed to further explore this association.

## Abbreviations

GTE: Green tea extract; RR: relative risk; 95% CI: 95% confidence intervals; HR: Hazard ratio; OR: Odds ratio; NOS: Newcastle-Ottawa Scale.

## Competing interests

The author’s declare that they have no competing interest.

## Author’s contributions

FY developed inclusion and exclusion criteria, drafted and revised the manuscript, contributed to the study design, analysis, and interpretation of the data. ZJ drafted the manuscript and contributed to the study design. HJ contributed to revising the manuscript, data analysis and interpretation of the data. CX developed inclusion and exclusion criteria, contributed to the data analysis and interpretation of the data. JT and TL contributed to the electronic search, hand-search of literature and the revision of the manuscript. JH contributed to the interpretation of the results and the conception of the study. All authors read and approved the final manuscript.

## Pre-publication history

The pre-publication history for this paper can be accessed here:

http://www.biomedcentral.com/1471-2407/14/197/prepub

## Supplementary Material

Additional file 1 The MOOSE checklist for this meta-analysis. Click here for file

## References

[B1] Food and Agriculture Organization of the United Nations Food balance sheets, 1994–1996 Available from: http://faostat3.fao.org/home/index.html#DOWNLOAD_STANDARD

[B2] Shimizu M Adachi S Masuda M Kozawa O Moriwaki H Cancer chemoprevention with green tea catechins by targeting receptor tyrosine kinases Mol Nutr Food Res 2011 55 6 832 843 10.1002/mnfr.201000622 21538846

[B3] Suzuki Y Miyoshi N Isemura M Health-promoting effects of green tea Proc Jpn Acad Ser B Phys Biol Sci 2012 88 3 88 101 10.2183/pjab.88.88 22450537 PMC3365247

[B4] Ahmad N Mukhtar H Green tea polyphenols and cancer: biologic mechanisms and practical implications Nutr Rev 1999 57 3 78 83 10101921 10.1111/j.1753-4887.1999.tb06927.x

[B5] Lambert JD Does tea prevent cancer? Evidence from laboratory and human intervention studies Am J Clin Nutr 2013 98 6 1667S 1675S 10.3945/ajcn.113.059352 24172300

[B6] World Cancer Research Fund/American Institute for Cancer Research Food, Nutrition, Physical Activity, and the Prevention of Cancer: a Global Perspective 2007 Washington DC: AICR

[B7] Choan E Segal R Jonker D Malone S Reaume N Eapen L Gallant V A prospective clinical trial of green tea for hormone refractory prostate cancer: an evaluation of the complementary/alternative therapy approach Urol Oncol 2005 23 2 108 113 10.1016/j.urolonc.2004.10.008 15869995

[B8] Nguyen MM Ahmann FR Nagle RB Hsu CH Tangrea JA Parnes HL Sokoloff MH Gretzer MB Chow HH Randomized, double-blind, placebo-controlled trial of polyphenon E in prostate cancer patients before prostatectomy: evaluation of potential chemopreventive activities Cancer Prev Res (Phila) 2012 5 2 290 298 10.1158/1940-6207.CAPR-11-0306 22044694 PMC3273617

[B9] Tsao AS Liu D Martin J Tang XM Lee JJ El-Naggar AK Wistuba I Culotta KS Mao L Gillenwater A Sagesaka YM Hong WK Papadimitrakopoulou V Phase II randomized, placebo-controlled trial of green tea extract in patients with high-risk oral premalignant lesions Cancer Prev Res (Phila) 2009 2 11 931 941 10.1158/1940-6207.CAPR-09-0121 19892663 PMC4243312

[B10] Sinha R Cross AJ Daniel CR Graubard BI Wu JW Hollenbeck AR Gunter MJ Park Y Freedman ND Caffeinated and decaffeinated coffee and tea intakes and risk of colorectal cancer in a large prospective study Am J Clin Nutr 2012 96 2 374 381 10.3945/ajcn.111.031328 22695871 PMC3396445

[B11] Yang G Zheng W Xiang YB Gao J Li HL Zhang X Gao YT Shu XO Green tea consumption and colorectal cancer risk: a report from the Shanghai Men’s Health Study Carcinogenesis 2011 32 11 1684 1688 10.1093/carcin/bgr186 21856996 PMC3246881

[B12] Ogunleye AA Xue F Michels KB Green tea consumption and breast cancer risk or recurrence: a meta-analysis Breast Cancer Res Treat 2010 119 2 477 484 10.1007/s10549-009-0415-0 19437116

[B13] Sing MF Yang WS Gao S Gao J Xiang YB Epidemiological studies of the association between tea drinking and primary liver cancer: a meta-analysis Eur J Cancer Prev 2011 20 3 157 165 10.1097/CEJ.0b013e3283447497 21403523

[B14] Sun CL Yuan JM Koh WP Yu MC Green tea, black tea and colorectal cancer risk: a meta-analysis of epidemiologic studies Carcinogenesis 2006 27 7 1301 1309 10.1093/carcin/bgl024 16638787

[B15] Wang XJ Zeng XT Duan XL Zeng HC Shen R Zhou P Association Between Green Tea and Colorectal Cancer Risk: A Meta-analysis of 13 Case–control Studies Asian Pac J Cancer Prev 2012 13 7 3123 3127 10.7314/APJCP.2012.13.7.3123 22994721

[B16] Zheng J Yang B Huang T Yu Y Yang J Li D Green tea and black tea consumption and prostate cancer risk: an exploratory meta-analysis of observational studies Nutr Cancer 2011 63 5 663 672 10.1080/01635581.2011.570895 21667398

[B17] Mao Q Lin Y Zheng X Qin J Yang K Xie L A meta-analysis of alcohol intake and risk of bladder cancer Cancer Causes Control 2010 21 11 1843 1850 10.1007/s10552-010-9611-9 20617375

[B18] Nagano J Kono S Preston DL Mabuchi K A prospective study of green tea consumption and cancer incidence, Hiroshima and Nagasaki (Japan) Cancer Causes Control 2001 12 6 501 508 10.1023/A:1011297326696 11519758

[B19] Ren JS Freedman ND Kamangar F Dawsey SM Hollenbeck AR Schatzkin A Abnet CC Tea, coffee, carbonated soft drinks and upper gastrointestinal tract cancer risk in a large United States prospective cohort study Eur J Cancer 2010 46 10 1873 1881 10.1016/j.ejca.2010.03.025 20395127 PMC2891563

[B20] Stroup DF Berlin JA Morton SC Olkin I Williamson GD Rennie D Moher D Becker BJ Sipe TA Thacker SB Meta-analysis of observational studies in epidemiology: a proposal for reporting. Meta-analysis Of Observational Studies in Epidemiology (MOOSE) group JAMA: j Am Med Assoc 2000 283 15 2008 2012 10.1001/jama.283.15.2008 10789670

[B21] Wells GA Shea B O’Connell D Peterson J Welch V Losos M Tugwell P The Newcastle-Ottawa Scale (NOS) for assessing the quality of nonrandomised studies in meta-analyses 2000 Ottawa, Canada: Department of Epidemiology and Community Medicine, University of Ottawa http://www.ohri.ca/programs/clinical_epidemiology/oxford.asp (accessed date Mar 16 2014)

[B22] Orsini N Bellocco RSG Generalized least squares for trend estimation of summarized dose–response data Stata J 2006 6 40 57

[B23] Greenland S Longnecker MP Methods for trend estimation from summarized dose–response data, with applications to meta-analysis Am J Epidemiol 1992 135 1301 1309 1626547 10.1093/oxfordjournals.aje.a116237

[B24] Greenland S Quantitative methods in the review of epidemiologic literature Epidemiol Rev 1987 9 1 30 3678409 10.1093/oxfordjournals.epirev.a036298

[B25] Ishitani K Lin J Manson JE Buring JE Zhang SM Caffeine consumption and the risk of breast cancer in a large prospective cohort of women Arch Intern Med 2008 168 18 2022 2031 10.1001/archinte.168.18.2022 18852405 PMC2574428

[B26] Lee KJ Inoue M Otani T Iwasaki M Sasazuki S Tsugane S Coffee consumption and risk of colorectal cancer in a population-based prospective cohort of Japanese men and women Int J Cancer 2007 121 6 1312 1318 10.1002/ijc.22778 17450527

[B27] Nechuta S Shu XO Li HL Yang G Ji BT Xiang YB Cai H Chow WH Gao YT Zheng W Prospective cohort study of tea consumption and risk of digestive system cancers: results from the Shanghai Women’s Health Study Am J Clin Nutr 2012 96 1056 1063 10/12 edn; 2012 10.3945/ajcn.111.031419 23053557 PMC3471195

[B28] Su LJ Arab L Tea consumption and the reduced risk of colon cancer – results from a national prospective cohort study Public Health Nutr 2002 5 3 419 425 12003653 10.1079/phn2001314

[B29] Dominianni C Huang WY Berndt S Hayes RB Ahn J Prospective study of the relationship between coffee and tea with colorectal cancer risk: the PLCO Cancer Screening Trial Br J Cancer 2013 109 5 1352 1359 10.1038/bjc.2013.434 23907431 PMC3778290

[B30] Montague JA Butler LM Wu AH Genkinger JM Koh WP Wong AS Wang R Yuan JM Yu MC Green and black tea intake in relation to prostate cancer risk among Singapore Chinese Cancer Causes Control 2012 23 10 1635 1641 10.1007/s10552-012-0041-8 22864870 PMC3695613

[B31] Oba S Shimizu N Nagata C Shimizu H Kametani M Takeyama N Ohnuma T Matsushita S The relationship between the consumption of meat, fat, and coffee and the risk of colon cancer: a prospective study in Japan Cancer lett 2006 244 2 260 267 10.1016/j.canlet.2005.12.037 16519996

[B32] Aune D Greenwood DC Chan DS Vieira R Vieira AR Navarro Rosenblatt DA Cade JE Burley VJ Norat T Body mass index, abdominal fatness and pancreatic cancer risk: a systematic review and non-linear dose–response meta-analysis of prospective studies Ann oncol : official j the Eur Soc Med Oncol / ESMO 2012 23 4 843 852 10.1093/annonc/mdr398 21890910

[B33] Dai Q Shu XO Li H Yang G Shrubsole MJ Cai H Ji B Wen W Franke A Gao YT Zheng W Is green tea drinking associated with a later onset of breast cancer? Ann Epidemiol 2010 20 1 74 81 10.1016/j.annepidem.2009.09.005 20006278 PMC2848451

[B34] DerSimonian R Laird N Meta-analysis in clinical trials Control Clin Trials 1986 7 3 177 188 10.1016/0197-2456(86)90046-2 3802833

[B35] Higgins JPT Green S Cochrane Handbook for Systematic Reviews of Interventions, Version 5.1.0 [updated March 2011] The Cochrane Collaboration 2011 Available from http://www.cochrane-handbook.org

[B36] Larsson SC Orsini N Coffee consumption and risk of stroke: a dose–response meta-analysis of prospective studies Am J Epidemiol 2011 174 9 993 1001 10.1093/aje/kwr226 21920945

[B37] Larsson SC Orsini N Wolk A Dietary magnesium intake and risk of stroke: a meta-analysis of prospective studies Am J Clin Nutr 2012 95 2 362 366 10.3945/ajcn.111.022376 22205313

[B38] Simons CC Leurs LJ Weijenberg MP Schouten LJ Goldbohm RA van den Brandt PA Fluid intake and colorectal cancer risk in the Netherlands Cohort Study Nutr Cancer 2010 62 3 307 321 10.1080/01635580903407098 20358468

[B39] Michels KB Willett WC Fuchs CS Giovannucci E Coffee, tea, and caffeine consumption and incidence of colon and rectal cancer J Natl Cancer Inst 2005 97 4 282 292 10.1093/jnci/dji039 15713963 PMC1909914

[B40] Hartman TJ Tangrea JA Pietinen P Malila N Virtanen M Taylor PR Albanes D Tea and coffee consumption and risk of colon and rectal cancer in middle-aged Finnish men Nutr Cancer 1998 31 1 41 48 10.1080/01635589809514676 9682247

[B41] Zheng W Doyle TJ Kushi LH Sellers TA Hong CP Folsom AR Tea consumption and cancer incidence in a prospective cohort study of postmenopausal women Am J Epidemiol 1996 144 2 175 182 10.1093/oxfordjournals.aje.a008905 8678049

[B42] Galanis DJ Kolonel LN Lee J Nomura A Intakes of selected foods and beverages and the incidence of gastric cancer among the Japanese residents of Hawaii: a prospective study Int J Epidemiol 1998 27 2 173 180 10.1093/ije/27.2.173 9602395

[B43] Inoue M Sasazuki S Wakai K Suzuki T Matsuo K Shimazu T Tsuji I Tanaka K Mizoue T Nagata C Tamakoshi A Sawada N Tsugane S Green tea consumption and gastric cancer in Japanese: a pooled analysis of six cohort studies Gut 2009 58 10 1323 1332 10.1136/gut.2008.166710 19505880

[B44] Suzuki Y Tsubono Y Nakaya N Koizumi Y Shibuya D Tsuji I Green tea and the risk of colorectal cancer: pooled analysis of two prospective studies in Japan J Epidemiol 2005 15 4 118 124 10.2188/jea.15.118 16141630 PMC7851069

[B45] Terry P Wolk A Tea consumption and the risk of colorectal cancer in Sweden Nutr Cancer 2001 39 2 176 179 10.1207/S15327914nc392_3 11759277

[B46] Higgins JP Thompson SG Quantifying heterogeneity in a meta-analysis Stat Med 2002 21 11 1539 1558 10.1002/sim.1186 12111919

[B47] Begg CB Mazumdar M Operating characteristics of a rank correlation test for publication bias Biometrics 1994 50 4 1088 1101 10.2307/2533446 7786990

[B48] Egger M Davey Smith G Schneider M Minder C Bias in meta-analysis detected by a simple, graphical test Br Med J 1997 315 7109 629 634 10.1136/bmj.315.7109.629 9310563 PMC2127453

[B49] Adebamowo CA Cho E Sampson L Katan MB Spiegelman D Willett WC Holmes MD Dietary flavonols and flavonol-rich foods intake and the risk of breast cancer Int J Cancer 2005 114 4 628 633 10.1002/ijc.20741 15609322

[B50] Allen NE Sauvaget C Roddam AW Appleby P Nagano J Suzuki G Key TJ Koyama K A prospective study of diet and prostate cancer in Japanese men Cancer Causes Control 2004 15 9 911 920 10.1007/s10552-004-1683-y 15577293

[B51] Key TJ Sharp GB Appleby PN Beral V Goodman MT Soda M Mabuchi K Soya foods and breast cancer risk: a prospective study in Hiroshima and Nagasaki Japan. Br J Cancer 1999 81 7 1248 1256 10.1038/sj.bjc.6690837 PMC2374337 10584890

[B52] Sauvaget C Lagarde F Nagano J Soda M Koyama K Kodama K Lifestyle factors, radiation and gastric cancer in atomic-bomb survivors (Japan) Cancer Causes Control 2005 16 7 773 780 10.1007/s10552-005-5385-x 16132787

[B53] Ellison LF Tea and other beverage consumption and prostate cancer risk: a Canadian retrospective cohort study Eur J Cancer Prev 2000 9 2 125 130 10830580 10.1097/00008469-200004000-00009

[B54] Hirvonen T Mennen LI de Bree A Castetbon K Galan P Bertrais S Arnault N Hercberg S Consumption of antioxidant-rich beverages and risk for breast cancer in French women Ann Epidemiol 2006 16 7 503 508 10.1016/j.annepidem.2005.09.011 16406814

[B55] Bhoo Pathy N Peeters P van Gils C Beulens JW van der Graaf Y Bueno-de-Mesquita B Bulgiba A Uiterwaal CS Coffee and tea intake and risk of breast cancer Breast Cancer Res Treat 2010 121 2 461 467 10.1007/s10549-009-0583-y 19847643

[B56] Boggs DA Palmer JR Stampfer MJ Spiegelman D Adams-Campbell LL Rosenberg L Tea and coffee intake in relation to risk of breast cancer in the Black Women’s Health Study Cancer Causes Control 2010 21 11 1941 1948 10.1007/s10552-010-9622-6 20680436 PMC3152948

[B57] Fagherazzi G Touillaud MS Boutron-Ruault MC Clavel-Chapelon F Romieu I No association between coffee, tea or caffeine consumption and breast cancer risk in a prospective cohort study Public Health Nutr 2011 14 7 1315 1320 10.1017/S1368980011000371 21466740

[B58] Ganmaa D Willett WC Li TY Feskanich D van Dam RM Lopez-Garcia E Hunter DJ Holmes MD Coffee, tea, caffeine and risk of breast cancer: a 22-year follow-up Int J Cancer 2008 122 9 2071 2076 10.1002/ijc.23336 18183588 PMC4186696

[B59] Goldbohm RA Hertog MG Brants HA van Poppel G van den Brandt PA Consumption of black tea and cancer risk: a prospective cohort study J Natl Cancer Inst 1996 88 2 93 100 10.1093/jnci/88.2.93 8537983

[B60] Iwasaki M Inoue M Sasazuki S Sawada N Yamaji T Shimazu T Willett WC Tsugane S Jphc FT Green tea drinking and subsequent risk of breast cancer in a population-based cohort of Japanese women Breast Cancer Res 2010 12 5 R88 10.1186/bcr2756 22889409 PMC3096981

[B61] Larsson SC Bergkvist L Wolk A Coffee and black tea consumption and risk of breast cancer by estrogen and progesterone receptor status in a Swedish cohort Cancer Causes Control 2009 20 10 2039 2044 10.1007/s10552-009-9396-x 19597749

[B62] Michels KB Holmberg L Bergkvist L Wolk A Coffee, tea, and caffeine consumption and breast cancer incidence in a cohort of Swedish women Ann Epidemiol 2002 12 1 21 26 10.1016/S1047-2797(01)00238-1 11750236

[B63] Suzuki Y Tsubono Y Nakaya N Koizumi Y Tsuji I Green tea and the risk of breast cancer: pooled analysis of two prospective studies in Japan Br J Cancer 2004 90 7 1361 1363 10.1038/sj.bjc.6601652 15054454 PMC2409667

[B64] Inoue M Kurahashi N Iwasaki M Shimazu T Tanaka Y Mizokami M Tsugane S Effect of coffee and green tea consumption on the risk of liver cancer: cohort analysis by hepatitis virus infection status Cancer Epidemiol Biomarkers Prev 2009 18 6 1746 1753 10.1158/1055-9965.EPI-08-0923 19505908

[B65] Ui A Kuriyama S Kakizaki M Sone T Nakaya N Ohmori-Matsuda K Hozawa A Nishino Y Tsuji I Green tea consumption and the risk of liver cancer in Japan: the Ohsaki Cohort study Cancer Causes Control 2009 20 10 1939 1945 10.1007/s10552-009-9388-x 19768563

[B66] Kikuchi N Ohmori K Shimazu T Nakaya N Kuriyama S Nishino Y Tsubono Y Tsuji I No association between green tea and prostate cancer risk in Japanese men: the Ohsaki Cohort Study Br J Cancer 2006 95 3 371 373 10.1038/sj.bjc.6603230 16804523 PMC2360636

[B67] Kurahashi N Sasazuki S Iwasaki M Inoue M Tsugane S Green tea consumption and prostate cancer risk in Japanese men: a prospective study Am J Epidemiol 2008 167 1 71 77 17906295 10.1093/aje/kwm249

[B68] Shafique K McLoone P Qureshi K Leung H Hart C Morrison DS Tea consumption and the risk of overall and grade specific prostate cancer: a large prospective cohort study of Scottish men Nutr Cancer 2012 64 6 790 797 10.1080/01635581.2012.690063 22697604

[B69] Geybels MS Verhage BA Arts IC van Schooten FJ Goldbohm RA van den Brandt PA Dietary flavonoid intake, black tea consumption, and risk of overall and advanced stage prostate cancer Am J Epidemiol 2013 177 12 1388 1398 10.1093/aje/kws419 23722011

[B70] Kang H Rha SY Oh KW Nam CM Green tea consumption and stomach cancer risk: a meta-analysis Epidemiol Health 2010 32 e2010001 21191454 10.4178/epih/e2010001 PMC2984861

[B71] Myung SK Bae WK Oh SM Kim Y Ju W Sung J Lee YJ Ko JA Song JI Choi HJ Green tea consumption and risk of stomach cancer: a meta-analysis of epidemiologic studies Int J Cancer 2009 124 3 670 677 10.1002/ijc.23880 18973231

[B72] Seely D Mills EJ Wu P Verma S Guyatt GH The effects of green tea consumption on incidence of breast cancer and recurrence of breast cancer: a systematic review and meta-analysis Integr Cancer Ther 2005 4 2 144 155 10.1177/1534735405276420 15911927

[B73] Sun CL Yuan JM Koh WP Yu MC Green tea, black tea and breast cancer risk: a meta-analysis of epidemiological studies Carcinogenesis 2006 27 7 1310 1315 10.1093/carcin/bgi276 16311246

[B74] Zhang X Albanes D Beeson WL van den Brandt PA Buring JE Flood A Freudenheim JL Giovannucci EL Goldbohm RA Jaceldo-Siegl K Jacobs EJ Krogh V Larsson SC Marshall JR McCullough ML Miller AB Robien K Rohan TE Schatzkin A Sieri S Spiegelman D Virtamo J Wolk A Willett WC Zhang SM Smith-Warner SA Risk of colon cancer and coffee, tea, and sugar-sweetened soft drink intake: pooled analysis of prospective cohort studies J Natl Cancer Inst 2010 102 11 771 783 10.1093/jnci/djq107 20453203 PMC2879415

[B75] Zhou Y Li N Zhuang W Liu G Wu T Yao X Du L Wei M Wu X Green tea and gastric cancer risk: meta-analysis of epidemiologic studies Asia Pac J Clin Nutr 2008 17 1 159 165 18364341

[B76] Platet N Cathiard AM Gleizes M Garcia M Estrogens and their receptors in breast cancer progression: a dual role in cancer proliferation and invasion Crit Rev Oncol Hematol 2004 51 1 55 67 10.1016/j.critrevonc.2004.02.001 15207254

[B77] Wu AH Arakawa K Stanczyk FZ Van Den Berg D Koh WP Yu MC Tea and circulating estrogen levels in postmenopausal Chinese women in Singapore Carcinogenesis 2005 26 5 976 980 15661801 10.1093/carcin/bgi028

